# Ketamine in Neurocritical Care: New Potentials and Perspectives

**DOI:** 10.7759/cureus.85456

**Published:** 2025-06-06

**Authors:** Rudin Domi, Alma Cani, Asead Abdyli, Gentian Huti, Stela Dodaj, Filadelfo Coniglione, Mirel Grada, Vojsava Leka, Majlinda Naco, Mustafa Bajraktari

**Affiliations:** 1 Anesthesiology, "Mother Teresa" University Hospital Center, Tirana, ALB; 2 Anesthesiology, American Hospital 3, Tirana, ALB; 3 Neurology, American Hospital 3, Tirana, ALB; 4 Surgical Sciences, "Our Lady of Good Counsel" University, Tirana, ALB; 5 Clinical Sciences and Translational Medicine, Tor Vergata University of Rome, Rome, ITA; 6 Neurosurgery, American Hospital 3, Tirana, ALB

**Keywords:** anticonvulsant, ketamine, neurocritical care, • neuroprotection, sedation

## Abstract

Ketamine is an intravenous hypnotic anesthetic that acts primarily by inhibiting N-methyl-D-aspartate (NMDA) receptors, leading to a range of effects, including hypnosis, analgesia, anticonvulsant activity, anti-inflammatory action, and neuroprotection. Initially, there were concerns that ketamine might elevate intracranial pressure. However, these worries have since been dispelled, leading to a renewed consideration of its role in neurocritical care. This evolving understanding has facilitated its increasing use in neurosurgical patients, both in the operating room and intensive care units, where it provides hemodynamic stability and neuroprotective benefits. Additionally, ketamine has shown promise in managing specific neurological conditions such as stroke and refractory seizures, further broadening its clinical applications. This review aims to provide a comprehensive clinical summary of ketamine’s usefulness in these settings.

## Introduction and background

Neurosurgical and neurocritical care patients often present significant challenges for anesthesiologists and intensivists. Managing these patients involves addressing conditions such as traumatic brain injury (TBI), ischemic stroke, intracranial hemorrhage, neuroprotection, seizures, and general critical care. A multidisciplinary team is essential, but treating the patient as a whole and individualizing therapy remain crucial. The primary goals of treatment include maintaining stable hemodynamic and respiratory function, ensuring adequate cerebral perfusion pressure (CPP), preventing further brain injury, providing adequate analgesia and sedation, and avoiding secondary complications such as seizures. These principles apply to both adult and pediatric patients [1].

Ketamine is a well-established anesthetic that has been used for decades across various clinical settings. Although it was once avoided in neurocritical care settings due to concerns about increasing intracranial pressure (ICP), current evidence no longer supports this theory. Ketamine acts on N-methyl-D-aspartate (NMDA) receptors, producing sedation, hypnosis, analgesia, and anticonvulsant effects. Although traditionally associated with the induction and maintenance of anesthesia, recent literature has increasingly highlighted its suitability in neurosurgical and neurocritical care settings. This renewed interest is driven by ketamine’s unique pharmacological profile, which includes the preservation of hemodynamic stability (often causing hypertension rather than the hypotension associated with other hypnotics), effective sedation and analgesia without significant respiratory depression, anti-inflammatory and neuroprotective properties, and utility in the treatment of refractory seizures. These characteristics make it particularly valuable in managing patients with neurological injuries, where maintaining CPP is essential. Moreover, ketamine’s applicability extends to the pediatric population, where its safety and efficacy have been demonstrated across a range of clinical scenarios, further supporting its role as a versatile agent in neuroanesthesia and critical care [2].

**Figure 1:**
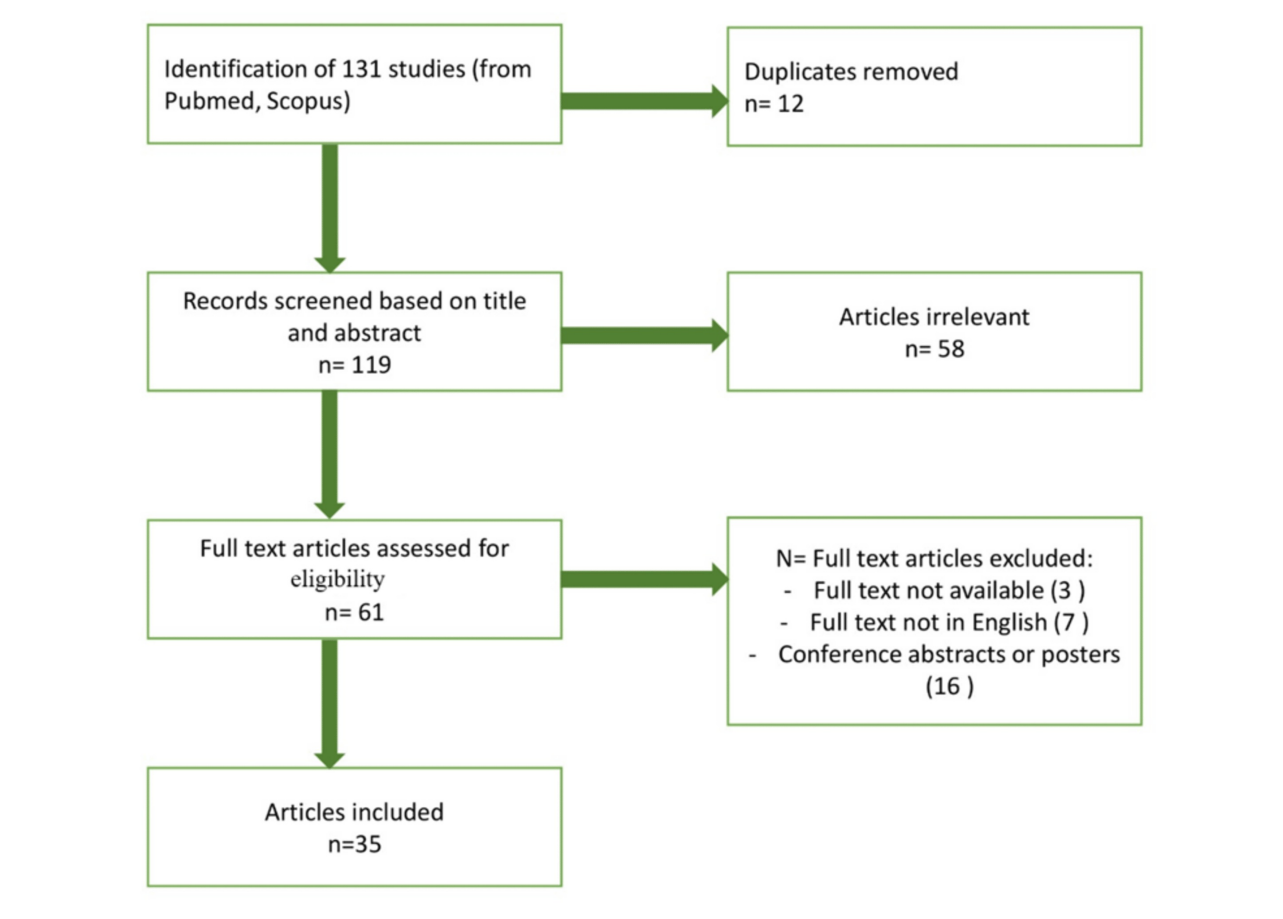
Inclusion criteria diagram

We searched PubMed and Scopus databases for recent literature on ketamine use in neurosurgery and neurocritical ill patients, using combined Medical Subject Headings (MeSH) search terms “ketamine,” “neurosurgery,” “neuroprotection,” “stroke,” “traumatic brain injury,” and “refractory epileptic status.” The research considered all papers from 2000 to January 2025. Narrative reviews, clinical trials, retrospective studies, case-control studies, and observational prospective studies were considered for possible inclusion. Two authors (R.D. and G.H.) independently conducted the research and analyzed all the articles. After completing the evaluation, they included 35 out of 131 papers in this narrative review (Figure 1).

The aim of this narrative review is to increase awareness of the safe use of ketamine in neurosurgery and neuro-intensive care units. The literature often presents limitations and controversies, so this narrative review tends to be a balanced one, despite our recommendation to consider ketamine a safe choice.

## Review

Ketamine use for TBI

During the intensive care unit (ICU) management of TBI, a key goal is preventing secondary brain injury, which can worsen outcomes. This requires addressing systemic and intracranial complications through strategies such as controlling pain and agitation, optimizing ventilation, monitoring respiratory effort, and managing seizures, temperature, glucose levels, and sodium balance [3]. Each of these factors plays a critical role in influencing cerebral metabolism and ICP dynamics.

A cornerstone of modern TBI care is the maintenance of adequate cerebral oxygenation and perfusion, which requires diligent support of cerebral hemodynamics and oxygen metabolism. This is typically achieved using analgesics and sedative agents that facilitate therapeutic sedation, reduce metabolic demand, and prevent abrupt fluctuations in ICP. Among the various anesthetic agents available, ketamine stands out due to its distinctive pharmacologic profile. It is the only hypnotic drug that combines analgesic, sedative, hypnotic, and dissociative properties within a single molecule. Moreover, it is characterized by a unique hemodynamic and respiratory profile that is particularly advantageous in the neurocritical care setting. Ketamine maintains hemodynamic stability, meaning it does not cause hypotension. This is particularly important in cases of elevated ICP, where hypotension should be strictly avoided. Ketamine helps preserve cerebral perfusion by maintaining systolic blood pressure above 100 mmHg. Ketamine acts primarily as an NMDA receptor antagonist, and emerging evidence suggests that it may have additional therapeutic benefits, such as the potential to suppress refractory seizures and provide neuroprotection in certain clinical contexts [4,5].

In the context of TBI, the overarching goal of sedation management is to maintain a delicate balance-providing sufficient analgesia and sedation to ensure patient comfort and minimize distress, while avoiding excessive sedation that could obscure neurological assessments or lead to complications. Light or minimal sedation lowers the risks associated with prolonged mechanical ventilation, such as ventilator-associated pneumonia, ICU-acquired weakness, and prolonged ICU stay. However, depending on the patient's condition or specific surgical interventions (e.g., external ventricular drainage), deep sedation may be necessary. Excessive sedation has also been associated with increased morbidity and mortality rates in critically ill patients [6]. Primary and secondary brain injuries are deeply interconnected through complex pathophysiological processes. For example, agitation in a TBI patient can lead to ventilator-patient asynchrony, which disrupts the effectiveness of mechanical ventilation and can precipitate episodes of hypercapnia, hypoxia, hypotension, or even hypotension. These respiratory and hemodynamic disturbances are detrimental to cerebral oxygenation and can intensify intracranial hypertension, thus compounding the injury to vulnerable brain tissue. Therefore, proper sedation and analgesia, alongside optimized ventilatory support, are essential to minimizing secondary insults and improving neurological outcomes.

Recent research has prompted a reevaluation of ketamine’s role in neurocritical care, challenging earlier concerns regarding its safety in patients with acute brain injury. Historically, ketamine was avoided in neurocritical patients due to the belief that it could elevate ICP, potentially exacerbating cerebral injury. However, a growing body of literature has refuted these concerns, demonstrating that ketamine does not produce sustained increases in ICP and may, in some cases, even reduce it. Furthermore, studies have shown that ketamine does not adversely affect cerebral oxygenation or impair cerebral autoregulation, which is critical for maintaining consistent cerebral perfusion in the face of fluctuating systemic pressures [7,8].

In addition to its hemodynamic neutrality, ketamine is gaining recognition for its potential neuroprotective effects. These include the inhibition of spreading depolarizations (SDs)-waves of electrical disturbance in the brain that are known to contribute to secondary brain injury. Ketamine also helps minimize systemic inflammatory responses and preserves cardiovascular and respiratory stability, without causing significant hypotension, hypoxia, and hypercapnia especially during endotracheal intubation. These properties make it a particularly appealing agent for sedating TBI patients who are critically ill and at risk of rapid clinical deterioration [9-12].

The comprehensive utility of ketamine in neurocritical care has been further supported by reviews and clinical studies. Notably, Zanza and colleagues conducted an in-depth narrative review of 11 studies examining the effects of ketamine in patients with acute brain injury. Among these studies, only two reported a slight increase in ICP, while another two documented a reduction. The remaining studies found no significant adverse impact on ICP. Based on these findings, the authors concluded that ketamine is a safe and effective anesthetic agent for use in TBI patients who require sedation and mechanical ventilation [13]. These results align with an emerging consensus in the field that ketamine, when used appropriately, can be integrated into sedation protocols for brain-injured patients with a favorable safety profile.

In summary, ketamine offers a multifaceted approach to sedation in the ICU setting for patients with TBI. Its ability to provide stable sedation and analgesia, support hemodynamic and respiratory function, inhibit potentially harmful neurophysiological phenomena, and possibly exert neuroprotective effects positions it as a valuable tool in the armamentarium of neurocritical care practitioners. As further research continues to elucidate its full range of effects and optimal usage strategies, ketamine may become increasingly central to evidence-based protocols for managing sedation and analgesia in TBI patients. Table 1 summarizes the possible positive effects of ketamine use in TBIs.

**Table 1:** Possible advantages of ketamine use in traumatic brain injury (original)

Advantages of ketamine use in traumatic brain injury
Effective analgesia
Hemodynamic stability
Minimize respiratory depression
Minimize spreading depolarization
Non-significant changes in intracranial pressure
Preserve cerebral oxygenation
Anticonvulsant properties

Ketamine's role in neuroprotection

Neuroprotection refers to clinical strategies aimed at preventing further brain injury. Ketamine appears to contribute to neuroprotection through its anti-inflammatory effects, anticonvulsant properties, and its ability to block cortical SDs-all of which may help limit secondary brain damage.

During ischemic events-particularly following resuscitation from cardiac arrest-a cascade of cellular and molecular events contributes to significant neuronal injury. One of the earliest disturbances involves the dysfunction of the sodium-potassium ATPase (Na⁺/K⁺ pump), which plays a crucial role in maintaining ionic gradients across the cell membrane. When this pump becomes impaired due to energy depletion, there is a resultant disruption of ion homeostasis. This leads to an accumulation of intracellular sodium and calcium, with the latter playing a pivotal role in downstream neurotoxicity.

The pathological increase in intracellular calcium concentration facilitates the aggregation of glutamate-containing vesicles at presynaptic terminals and their subsequent exocytosis into the synaptic cleft. This excess glutamate overactivated postsynaptic ionotropic receptors, particularly NMDA receptors, leading to a phenomenon known as excitotoxicity. Excitotoxic damage is characterized by further calcium influx, mitochondrial dysfunction, and activation of proteolytic enzymes, ultimately culminating in neuronal cell death.

A secondary phase of injury, termed reperfusion injury, occurs upon the restoration of blood flow. While oxygen delivery resumes, it is often not matched by efficient oxygen utilization due to ongoing mitochondrial dysfunction. This mismatch exacerbates oxidative stress through the generation of reactive oxygen species (ROS), further promoting lipid peroxidation, DNA fragmentation, and the activation of apoptotic pathways. Together, these events contribute to delayed neuronal death and worsen neurological outcomes [14,15].

The potential neuroprotective role of ketamine, an NMDA receptor antagonist, remains a subject of ongoing debate. Despite its established anesthetic and analgesic properties, its effect on neural tissue under ischemic or traumatic conditions has yet to reach clinical consensus. Nevertheless, several proposed mechanisms highlight its potential as a neuroprotective agent.

Ketamine exhibits a dose-dependent blockade of NMDA receptors, thereby reducing calcium influx and mitigating excitotoxic neuronal injury. It also appears to suppress presynaptic glutamate release and may modulate glutamatergic transmission more broadly. Furthermore, by reducing intracellular calcium accumulation, ketamine could potentially attenuate downstream pathways that lead to neuronal apoptosis and necrosis.

Although human clinical data are limited, preclinical investigations offer supportive evidence. In animal models-particularly studies employing transient carotid artery occlusion to mimic cerebral ischemia-ketamine administration has been associated with reduced hippocampal damage. These effects appear most prominent at higher doses (10-20 mg/kg), suggesting a dose threshold may be necessary for therapeutic efficacy [14,15].

Additional preclinical studies have reported that ketamine reduces the extent of the ischemic zone and limits the progression of infarct size in models of experimental brain injury, possibly through a combination of anti-inflammatory, antiapoptotic, and vasodilatory effects [16,17].

Since 2020, several significant studies have advanced the understanding of ketamine’s neuroprotective profile. For instance, Guliano et al. investigated using a canine model of cardiopulmonary bypass, a procedure known to increase the risk of cerebral ischemia and postoperative cognitive dysfunction. Their findings revealed that animals treated with ketamine displayed fewer neurobehavioral abnormalities, along with significantly lower levels of both inflammatory markers (such as IL-6 and TNF-α) and neuronal injury biomarkers, suggesting a potential role for ketamine in perioperative neuroprotection [18]. In a randomized control trial involving human subjects, administration of 0.5 mg/kg ketamine led to improved neurocognitive outcomes in the intervention group. Specifically, patients demonstrated superior performance in both verbal and non-verbal memory assessments, along with decreased systemic inflammation as indicated by reduced C-reactive protein (CRP) levels [19]. These findings underscore ketamine’s ability to modulate neuroinflammatory responses and support cognitive preservation following ischemic insults. Another promising mechanism by which ketamine may confer neuroprotection is through the suppression of SDs-waves of near-complete depolarization that propagate through brain tissue following TBI or stroke. SDs are associated with the exacerbation of neuronal injury, particularly in metabolically compromised regions. Recent studies have shown that ketamine can effectively inhibit these depolarization cascades, likely due to its NMDA antagonism and dampening of cortical excitability [20]. In a comprehensive narrative review, Bell summarized the multifaceted actions of ketamine, suggesting that its protective properties extend beyond mere NMDA antagonism. According to this review, ketamine exhibits anti-inflammatory, antiexcitotoxic, and antimicrothrombotic effects, each of which may contribute synergistically to the preservation of ischemic brain tissue [21]. By modulating microglial activation, inhibiting pro-inflammatory cytokines, and improving microvascular flow, ketamine may limit both primary and secondary injury mechanisms following ischemic or traumatic events. Table 2 summarizes the proposed neuroprotective mechanisms of ketamine.

**Table 2:** Proposed neuroprotective mechanisms of ketamine (original)

Neuroprotective mechanisms of ketamine
Blocks calcium intracellular influx and excitotoxic phenomena
Reduces presynaptic glutamate release
Reduces inflammation
Exerts antimicrothrombotic properties
Reduces the ischemic damaged zone
Inhibits spreading depolarization

Ketamine use for neuroanesthesia and stroke units

The longstanding belief that ketamine increases ICP remains a topic of debate, as recent evidence has yet to confirm this paradox. The primary anesthetic goals for neurosurgical procedures include brain relaxation, maintaining CPP stability, preserving hemodynamic parameters, and preventing hypotension [22]. Two main techniques are commonly employed to achieve these objectives: total intravenous anesthesia (TIVA) and inhalation anesthesia following intravenous induction.

During anesthesia induction for intracranial tumors particularly in cases with increased ICP or altered mental status, the anesthesiologist must avoid hypotension, hypoxia, and hypercapnia. Hypotension can compromise CPP, while hypercapnia can exacerbate ICP through excessive cerebral vasodilation.

Concerns regarding ketamine's effects on ICP stem from early studies conducted in non-ventilated patients, where elevations in blood pressure and cerebral blood flow could translate to increased ICP. However, modern studies using controlled ventilation and adequate sedation using ketamine have found no consistent evidence of harmful ICP elevation. In fact, ketamine’s favorable hemodynamic profile-marked by its sympathomimetic properties-makes it especially valuable in preventing peri-induction hypotension, a common and dangerous occurrence in neurocritical ill patients. Several contemporary investigations have explored ketamine’s role during anesthetic induction for neurosurgery. Their findings generally indicate that ketamine can maintain or even improve cerebral perfusion without significantly increasing ICP. For example, when administered to patients with pre-existing elevated ICP, ketamine was shown to preserve hemodynamic parameters and avoid respiratory depression, both of which are crucial in mitigating secondary brain injury [23].

Grathwohl et al. conducted a comparative analysis involving 252 patients undergoing neurosurgical procedures, divided into three groups: TIVA without ketamine, TIVA with ketamine, and inhalational anesthesia. The study aimed to determine if any anesthetic regimen led to superior outcomes. Despite the robust sample size, the authors were unable to demonstrate statistically significant differences in patient outcomes between the groups, suggesting that the inclusion of ketamine does not negatively influence overall neurological prognosis [24].

A broader evaluation of the literature was conducted by Cohen et al. in a systematic review comprising 11 studies. The review concluded that ketamine, when used as part of anesthetic or induction protocols in adult patients-particularly in emergency department settings-did not increase ICP or worsen neurological outcomes. Interestingly, a transient decrease in ICP was noted in several studies, further dispelling the myth of ketamine-induced ICP elevation. Additionally, no adverse impact on CPP, overall neurological function, or mortality was found compared to other commonly used induction agents such as etomidate or propofol [25].

The principles of managing neurologically compromised patients in the ICU parallel those in the operating room but include additional considerations such as prolonged sedation, ventilation management, and prevention of delayed complications. In patients with subarachnoid hemorrhage (SAH), for example, vasospasm remains a leading cause of delayed ischemic deficits. A growing body of evidence supports ketamine’s ability to suppress cortical SDs, which are implicated in the pathophysiology of vasospasm-related secondary injury [26].

Further expanding on its potential neuroprotective benefits, ketamine has also been investigated in the acute management of ischemic stroke. Some emerging studies propose that combining ketamine with tissue plasminogen activator (tPA) may reduce the extent of reperfusion injury and subsequent ischemic damage, though more rigorous clinical trials are needed to confirm this strategy [27].

In the pediatric ICU, where sedation and ICP management are particularly challenging, ketamine has shown promising results. One study involving 30 ventilated pediatric patients with elevated ICP documented a consistent reduction in ICP after repeated ketamine administration while maintaining stable hemodynamics. These findings affirm that ketamine may be safe and effective for managing intracranial hypertension in children [28].

In their retrospective observational study, Dengler et al. analyzed data from 44 patients admitted to neurocritical care units with severe TBI. All patients were under mechanical ventilation and had ICP monitoring. The study found that the administration of ketamine boluses was associated with a significant reduction in ICP and an increase in CPP. Specifically, the median reduction in ICP after ketamine administration was 3.5 mmHg, and the increase in CPP was 2 mmHg, both statistically significant changes (p < 0.001) [29]. Ketamine as a sedative agent in neurocritical patients remains an intriguing subject. Literature supports ketamine as a viable option. Von der Brelie et al. administered ketamine to 41 out of 65 patients enrolled in their study following ICU admission for SAH. They observed a reduction in ICP in 92.7% of patients and, notably, a 53.6% decrease in vasopressor requirements [30]. A comprehensive review by Sethuraman et al. provides a synthesized perspective on ketamine’s role in neurological and neurosurgical care settings, both in the operating room and ICU. Their work outlines a broad range of beneficial effects, including maintenance of CPP without causing ICP spikes; sympathomimetic support, which minimizes the risk of hypotension during induction; respiratory preservation, avoiding hypoventilation-related hypercapnia; inhibition of SDs, potentially reducing delayed cerebral ischemia; and anti-inflammatory and neuroprotective effects, enhancing patient outcomes in traumatic and ischemic injuries [31]. Table 3 presents several possible positive effects of ketamine use in neurological/neurosurgical settings.

**Table 3:** Possible positive effects of ketamine use in neurological/neurosurgical settings (original)

Possible positive effects of ketamine use in neurological/neurosurgical patients
Analgesia
Hemodynamic stability
Anti-inflammatory
Sedation
Anticonvulsant

Ketamine as rescue therapy in refractory epileptic status

Ketamine has been increasingly explored as a potential therapeutic option for the management of status epilepticus (SE), particularly in cases that progress to refractory SE (RSE) and super-RSE (SRSE), where conventional treatments fail. As a third-line agent, ketamine offers a mechanistically distinct approach from standard first- and second-line therapies. Its principal mechanism of action is mediated through the non-competitive antagonism of NMDA receptors. This is particularly relevant in the context of prolonged seizure activity, during which neurochemical changes occur, including the downregulation of gamma-aminobutyric acid (GABA) receptors and upregulation or increased activation of NMDA receptors. These alterations are believed to contribute significantly to the pharmaco-resistance observed with GABAergic agents such as benzodiazepines, which often lose efficacy as seizures persist. Thus, targeting excitatory glutamatergic transmission via NMDA receptor inhibition provides a rational strategy for the management of RSE [32].

Despite the biological plausibility and pharmacologic rationale, the clinical evidence supporting the use of ketamine in SE remains limited. In a systematic review conducted by Rosati et al., no randomized controlled trials (RCTs) were identified that evaluated ketamine’s efficacy in this setting. Instead, their review relied exclusively on case reports and small observational studies. Nonetheless, the authors concluded that ketamine appeared to be a promising and potentially valuable option in the treatment of RSE, particularly when used as part of a multimodal therapeutic regimen [33].

Further support for ketamine’s utility comes from smaller case series and retrospective analyses. One such case series documented the use of ketamine infusion in 11 patients experiencing refractory epileptic crises. In this cohort, ketamine not only facilitated the successful termination of seizures but was also associated with improved hemodynamic parameters, including a reduction in vasopressor requirements-an important consideration in critically ill patients with compromised cardiovascular status [34].

More substantial clinical data were presented in a multicenter retrospective study involving 58 patients diagnosed with RSE. This study reported that permanent seizure control was achieved in 57% of treatment episodes following ketamine administration. These findings underscore ketamine’s potential as an effective adjunctive therapy in RSE, particularly when conventional antiepileptic agents have failed to provide seizure control [35]. While prospective, randomized data are still lacking, current evidence suggests that ketamine may represent a viable and beneficial component of third-line therapy in the management of refractory seizures.

Limitations

There are several limitations to this paper. First, the use of ketamine in neurocritical patients remains a topic of ongoing debate. More time and research are needed to fully understand its safety profile in this population. Second, there are no specific clinical guidelines on this issue-only individual studies, including reviews, meta-analyses, and RCTs. Third, there is a lack of standardized data on ketamine use for these purposes, and the existing literature is often inconsistent. This may be due to the sporadic use of ketamine, influenced by outdated concerns about its potential to increase ICP.

## Conclusions

Ketamine is a dissociative anesthetic that works primarily by blocking NMDA receptors, leading to effects such as sedation, analgesia, neuroprotection, and antiepileptic properties. Although it was once thought to raise ICP, recent evidence suggests it may be safe for patients with brain injuries such as trauma and stroke. It supports both systemic and cerebral hemodynamics by stimulating the sympathetic nervous system, helping to maintain cerebral perfusion. Ketamine may also reduce harmful cortical SDs linked to secondary brain injury. These properties make it a valuable option for managing severe neurological conditions, particularly when other agents pose hemodynamic risks.
